# Albumin: a novel biomarker for predicting intraoperative hypothermia in HSCR

**DOI:** 10.1080/07853890.2025.2540019

**Published:** 2025-07-30

**Authors:** Xiaohui Huang, Mobai Ren, Junrong Pan, Ene Huang, Yanhong Li, Donghao Guo, Junjie Wang

**Affiliations:** a Department of Operating Room, Quanzhou Women’s and Children’s Hospital, Quanzhou, China; b School of Clinical Medicine, Shanghai Renji Hospital, School of Medicine, Shanghai Jiaotong University, Shanghai, China; c Department of Pediatric Endocrinology, Quanzhou Women’s and Children’s Hospital, Quanzhou, China; d Department of Cardiology, Shanghai Renji Hospital, School of Medicine, Shanghai Jiaotong University, Shanghai, China

**Keywords:** Intraoperative hypothermia, Hirschsprung’s disease, albumin

## Abstract

**Background:**

Intraoperative hypothermia is a significant life-threatening emergency during surgery in patients with Hirschsprung’s disease (HSCR). The aim of this study is to explore the risk factors and predictors of intraoperative hypothermia in HSCR patients.

**Methods:**

This cohort comprised 85 patients with HSCR who underwent surgery at Quanzhou Children’s Hospital and the patients were divided into the intraoperative non-hypothermia group and the intraoperative hypothermia group. The study compared the characteristics of two groups and used univariate and multiple logistic regression analyses to assess the potential risk factors for intraoperative hypothermia. Models were adjusted for covariates, and interaction terms were evaluated for albumin (ALB) and intraoperative hypothermia. Subgroup analysis included stratification by sex and age. ROC analysis was applied to determine the optimal threshold for ALB.

**Results:**

In this retrospective study, 71 patients had intraoperative non-hypothermia and 14 patients had intraoperative hypothermia (83.5% versus 16.5%). Comparing the clinical characteristics between two groups, baseline core temperature, ALB and alkaline phosphatase (*p* < .001, *p* = .001 and *p* = .036, respectively) showed significant differences. Univariate logistic regression showed that baseline core temperature (OR = 0.001, 95%CI = 0.000–0.024, *p* < .001), ALB (OR = 0.820, 95%CI = 0.679–0.972, *p* = .028) and gamma-glutamyl transferase (OR = 1.017, 95%CI = 1.001–1.035, *p* = .043) had significant associations with intraoperative hypothermia. Multiple logistic regression showed that both ALB (OR = 0.782, 95%CI = 0.611–0.965, *p* = .031) and baseline core temperature (OR = 0.001, 95%CI = 0.000–0.019, *p* < .001) were negatively associated with intraoperative hypothermia. The relationship between ALB and intraoperative hypothermia remained significant after adjusting for covariates. ROC analysis identified 41.45 g/L as the optimal threshold of ALB for predicting intraoperative hypothermia, with a sensitivity of 85.71% and a specificity of 64.79%.

**Conclusion:**

ALB is an independent risk factor for intraoperative hypothermia in HSCR patients. Further investments are required to explore its mechanisms.

## 1. Introduction

Hirschsprung’s disease (HSCR) is a rare congenital gastrointestinal disorder characterized by the absence of enteric ganglia in a segment of the colon, resulting in impaired peristalsis and subsequent intestinal obstruction. HSCR affects approximately 1 in 5000 newborns with a male-to-female ratio of 4:1 [1,2]. Patients with HSCR typically present with delayed passage of meconium in newborns, along with persistent constipation and abdominal distension in infants and children. They may also experience vomiting, malnutrition and developmental delays [3]. The standard treatment for HSCR involves surgical resection of the aganglionic segment of bowel and anastomosis of proximal bowel to the anus [4]. However, surgery for HSCR is often complex [3], and emergencies can arise during and after the operation, such as intraoperative hypothermia, infection and Hirschsprung-associated enterocolitis [5,6].

Accidental intraoperative hypothermia, a common complication among surgical patients defined as core temperature below 36 °C [7], can lead to slowed metabolism [8], postoperative cardiovascular events [9], perioperative haemorrhage [10] and postoperative infection [11]. These complications may result in delayed anaesthesia recovery [12] and prolonged hospital stays [13–15]. The prolonged surgical duration and intricate dissection in HSCR surgeries significantly increase patients’ exposure to the environment, potentially intensifying the risk of hypothermia [16,17]. As a result, it is imperative to comprehend the factors that contribute to intraoperative hypothermia in HSCR to enhance disease management.

Despite numerous studies examining the risk factors of hypothermia during surgeries [15,18–23], there is a conspicuous dearth of research specifically focused on intraoperative hypothermia in HSCR. Notably, few studies have yet incorporated relevant laboratory indicators to assess their influence on this phenomenon [13,23]. By bridging this gap, this study aims to delve deeper into the relevant factors associated with hypothermia during HSCR surgeries, and help improve the prevention and management protocols for intraoperative hypothermia in HSCR patients.

## 2. Method

### 2.1. Patients and ethics

This retrospective study was carried out at the Quanzhou Children’s hospital (Quanzhou, China) after being approved by the Ethics Committee. A total of 85 patients who underwent surgery under general anaesthesia from 11 December 2017 to 3 August 2024 were enrolled. The characteristics and perioperative information of these patients were meticulously gathered and subjected to rigorous analysis. The data was sourced from electronic medical records, with strict adherence to the principle of de-identification to ensure patient confidentiality. Given the retrospective and anonymized nature of the data collected, the written informed consent from the patients was deemed unnecessary.

The study exclusively encompassed patients diagnosed with Hirschsprung’s Disease (HSCR) in accordance with the 2017 guidelines established by The Group of Anorectum, The Group of Neonatology, and the Society of Pediatric Surgery, under the auspices of the Chinese Medical Association [3]. Notably, patients who did not undergo surgical intervention at the Quanzhou Children’s Hospital were excluded from the study.

### 2.2. Anaesthesia

Once patients had entered the operating room, we conducted routine monitoring procedures, including haemoglobin oxygen saturation (SpO_2_), electrocardiogram (ECG), and either noninvasive blood pressure (NIBP) or invasive blood pressure (IBP). All surgeries were performed under tracheal intubation, utilizing the induction anaesthesia medications that comprised 1% propofol, sufentanil, recouronium bromide, midazolam and sevoflurane [24].

### 2.3. Core temperature measurement

The operating room temperature was set at 22–24 °C, with humidity maintained within 50–60%. All patients were passively warmed using standardized medical warming blankets. Body temperature was continually monitored through nasopharyngeal temperature detection, with automatically recording the child’s nasopharyngeal temperature every 15 min [17,22]. The primary outcome was inadvertent intraoperative hypothermia, defined as a core temperature below 36 °C at any time during the surgery [25]. Patients were subsequently divided into two groups based on the occurrence of intraoperative hypothermia: hypothermia group and the non-hypothermia group.

### 2.4. Statistical analysis

In order to investigate the potential risk factors contributing intraoperative hypothermia, we compared demographic characteristics (sex and age) and potential predictors (weight, body surface area, laparoscopic surgery, duration of anaesthesia, operation duration, intraoperative fluid intake, intraoperative irrigation, intraoperative blood loss, baseline core temperature, prothrombin time [PT], activated partial thromboplastin time [APTT], fibrinogen [FG], albumin [ALB], alanine aminotransferase [ALT], aspartate aminotransferase [AST], alkaline phosphatase [ALP], gamma-glutamyl transferase [GGT], lactate dehydrogenase [LDH], white blood cell [WBC], neutrophil percentage [N%], lymphocyte percentage [L%], haemoglobin [HGB], platelet [PLT]) between the hypothermia group and the non-hypothermia group. No variables in this study had missing values exceeding 20%. Missing data were addressed using the Multiple Imputation by Chained Equations (MICE) package in R. Continuous variables were reported as mean ± standard deviation, while categorical variables were presented as frequencies in percentage form. For continuous data, independent samples t-tests were used for comparison, while categorical data were analysed using chi-square tests. Univariate and multiple logistic regressions were used to evaluate the clinical characteristics and potential risk factors for intraoperative hypothermia. Models were adjusted for covariates, and interaction terms were evaluated for ALB and intraoperative hypothermia. Subgroup analysis included stratification by sex and age. Statistical analysis was performed using R version 4.4.0 (https://www.r-project.org/), with a *p* value of <.05 considered statistically significant.

## 3. Result

### 3.1. Comparison of the clinical characteristics of the two groups

In this retrospective study, a total of 85 patients who had undergone surgical intervention for HSCR were enrolled. As depicted in Table 1, patients with HSCR were categorized into two groups based on the occurrence of intraoperative hypothermia. Specifically, 71 patients (83.5%) did not experience intraoperative hypothermia, whereas 14 patients (16.5%) did. HSCR patients with intraoperative non-hypothermia exhibited significantly elevated baseline core temperature level (36.6 ± 0.261 vs. 36.2 ± 0.274, *p* < .001) and higher level of ALB (42.5 ± 3.77 vs. 40.1 ± 1.89, *p* = .001). Conversely, the ALP of HSCR patients in the intraoperative non-hypothermia group was significantly lower than that in the intraoperative hypothermia group (274 ± 96.0 vs. 328 ± 80.4, *p* = .036). Additionally, their neutrophil count in the intraoperative non-hypothermia group displayed marginally lower trend compared to the intraoperative hypothermia group (27.3 ± 12.5 vs. 20.3 ± 13.7, *p* = .091). However, no statistically significant differences were observed between the two groups regarding intraoperative blood loss, anaesthesia duration, APTT or other parameters (all *p* > .1).

**Table 1.** Baseline characteristics of HSCR patients according to the presence of intraoperative hypothermia.

Characteristics	Non-hypothermia group (*N* = 71)	Hypothermia group (*N* = 14)	*p* Value
Sex			
Female	13 (18.3%)	1 (7.1%)	.525
Male	58 (81.7%)	13 (92.9%)	
Age (months)	16.2 ± 29.4	10.0 ± 24.8	.415
Weight (kg)	8.69 ± 4.78	7.14 ± 4.70	.276
Body surface area (m^2^)	0.404 ± 0.167	0.350 ± 0.165	.276
Laparoscopic surgery			
No	20 (28.2%)	6 (42.9%)	.44
Yes	51 (71.8%)	8 (57.1%)	
Duration of anaesthesia (minutes)	286 ± 115	291 ± 112	.891
Operation duration (minutes)	225 ± 110	215 ± 90.5	.739
Intraoperative fluid intake (ml)	350 ± 298	299 ± 316	.586
Intraoperative irrigation			
No	65 (91.5%)	13 (92.9%)	1
Yes	6 (8.5%)	1 (7.1%)	
Intraoperative blood loss (ml)	8.59 ± 12.2	7.86 ± 7.90	.776
Baseline core temperature (°C)	36.6 ± 0.261	36.2 ± 0.274	<.001***
Intraoperative hypothermia			
No	71 (100%)	0 (0%)	<.001***
Yes	0 (0%)	14 (100%)	
PT (s)	11.3 ± 0.889	11.5 ± 0.728	.234
APTT (s)	31.9 ± 6.92	32.7 ± 4.85	.594
FG (g/L)	2.00 ± 0.491	2.12 ± 0.592	.472
ALB (g/L)	42.5 ± 3.77	40.1 ± 1.89	.001**
ALT (U/L)	30.5 ± 18.5	34.1 ± 18.8	.518
AST (U/L)	46.2 ± 16.9	49.8 ± 21.1	.555
ALP (U/L)	274 ± 96.0	328 ± 80.4	.036*
GGT (U/L)	29.3 ± 26.7	50.7 ± 44.4	.101
LDH (U/L)	279 ± 53.2	284 ± 54.2	.753
WBC (10^9^/L)	9.77 ± 2.89	9.50 ± 2.51	.724
Neutrophil (%)	27.3 ± 12.5	20.3 ± 13.7	.091
Lymphocyte (%)	60.0 ± 12.9	63.9 ± 13.1	.325
HGB (g/L)	115 ± 11.2	111 ± 8.97	.218
PLT (10^9^/µL)	410 ± 134	441 ± 105	.349

*Notes:* Data are presented as mean ± *SD*. *Denotes statistically differences between the two groups. *<.05; **<.01; ***<.001.

### 3.2. Analysis of potential risk factors for HSCR

As depicted in Table 2, the univariate logistic regression analysis revealed a significant association between a lower baseline core temperature (OR = 0.001, 95%CI = 0.000–0.024, *p* < .001) and ALB levels (OR = 0.820, 95%CI = 0.679–0.972, *p* = .028) with a decreased risk of intraoperative hypothermia. Contrastingly, an elevated GGT (OR = 1.017, 95%CI = 1.001–1.035, *p* = .043) was correlated with higher risk of intraoperative hypothermia.

**Table 2.** Univariate logistic regression analysis on the risk factors for HSCR.

Variable	Odds Ratio (95% CI)	*p* Value
Sex	2.914 (0.507, 55.267)	.323
Month old	0.989 (0.947, 1.011)	.471
Weight	0.900 (0.713, 1.046)	.280
Body surface area	0.050 (0.000, 3.629)	.280
Laparoscopic surgery	0.523 (0.161, 1.765)	.281
Duration of anaesthesia	1.000 (0.995, 1.005)	.890
Operation duration	0.999 (0.992, 1.004)	.764
Intraoperative fluid intake	0.999 (0.996, 1.001)	.562
Intraoperative irrigation	0.833 (0.042, 5.471)	.871
Intraoperative blood loss	0.994 (0.906, 1.039)	.828
Baseline core temperature	0.001 (0.000, 0.024)	<.001***
PT	1.922 (1.021, 3.798)	.048*
APTT	1.019 (0.932, 1.110)	.669
FG	1.637 (0.546, 4.725)	.362
ALB	0.820 (0.679, 0.972)	.028*
ALT	1.010 (0.979, 1.039)	.505
AST	1.012 (0.979, 1.044)	.481
ALP	1.006 (1.000, 1.012)	.058
GGT	1.017 (1.001, 1.035)	.043
LDH	1.002 (0.991, 1.012)	.744
WBC	0.965 (0.770, 1.180)	.742
Neutrophil	0.937 (0.864, 0.996)	.072
Lymphocyte	1.027 (0.980, 1.090)	.309
HGB	0.970 (0.915, 1.023)	.274
PLT	1.002 (0.997, 1.006)	.417

*Note:* *<.05; **<.01; ***<.001.

In the stepwise multiple analysis presented in Table 3, the results of logistic regression analyses indicated that ALB (OR = 0.782, 95%CI = 0.611–0.965, *p* = .031) and baseline core temperature (OR = 0.001, 95%CI = 0.000–0.019, *p* < .001) had a significant association with intraoperative hypothermia.

**Table 3.** Multiple logistic regression analysis of predictors for HSCR.

Predictor	Odds ratio (95% CI)	*p* Value
ALB	0.782 (0.611, 0.965)	.031*
Baseline core temperature	0.001 (0.000, 0.019)	<.001***

*Note:* *<.05; **<.01; ***<.001.

### 3.3. The independent effect of albumin on the intraoperative hypothermia

Table 4 shows the correlation between intraoperative hypothermia and serum ALB. In model 1, without adjustment, individuals with lower serum ALB levels had a greater risk of intraoperative hypothermia (OR = 0.820, 95%CI = 0.679–0.972, *p* = .028). In model 2, baseline core temperature was incorporated into the analysis, and ALB continued to demonstrate a significant association with intraoperative hypothermia (OR = 0.782, 95%CI = 0.611–0.965, *p* = .031). Subsequently, in model 3 with additional adjustment for sex and age, this correlation remained significant (OR = 0.732, 95%CI = 0.532–0.944, *p* = .029). Finally, we further included ALP and GGT in model 4, and the negative association between intraoperative hypothermia and serum ALB persisted (OR = 0.734, 95%CI = 0.523–0.969, *p* = .044). Collectively, the findings from all four models indicate that ALB possesses a robust predictive capability for intraoperative hypothermia in HSCR patients.

**Table 4.** The association between serum levels of ALB and intraoperative hypothermias among HSCR patients.

Predictor	Model	Odds ratio (95% CI)	*p* Value
ALB	Model 1	0.820 (0.679, 0.972)	.028*
ALB	Model 2	0.782 (0.611, 0.965)	.031*
ALB	Model 3	0.732 (0.532, 0.944)	.029*
ALB	Model 4	0.734 (0.523, 0.969)	.044*

*Notes:* *<.05; **<.01; ***<.001. Model 1: unadjusted. Model 2: adjusted for baseline core temperature. Model 3: adjusted for factors in model 2 plus sex and age. Model 4: adjusted for factors in model 3 plus ALP and GGT.

### 3.4. Subgroup analysis

Subgroup analysis was conducted to further investigate the association of patients’ serum ALB levels with intraoperative hypothermia among different groups. As shown in Table 5, there is no significant association between different groups of age/sex among HSCR patients (interaction *p* value > .05). These results confirmed the negative association between serum ALB levels and the risk of intraoperative hypothermia in different subgroups.

**Table 5.** Stratified subgroup analysis.

Subgroup	Odds ratio (95% CI)	*p* Value	Interaction *p* value
Age (months)			
≤12	0.851 (0.696, 1.018)	.091	.746
>12	0.764 (0.355, 1.480)	.417	
Sex			
Male	0.828 (0.680, 0.985)	.043*	.770
Female	0.949 (0.306, 2.471)	.911	

*Note:* *<.05; **<.01; ***<.001.

### 3.5. Analysis of albumin threshold for predicting intraoperative hypothermia

**Figure 1.**
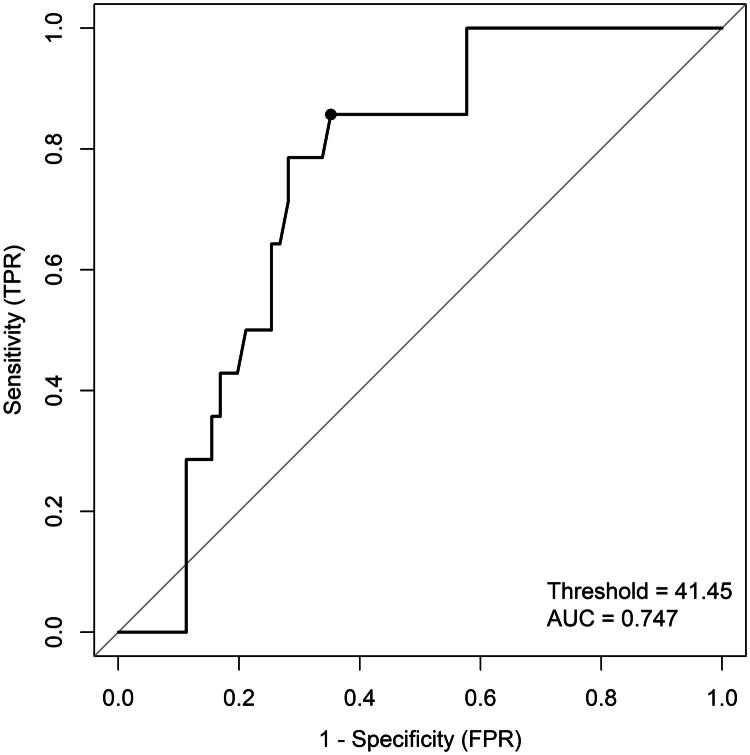
ROC curve of the diagnostic accuracy of ALB.

ROC analysis was further applied to explore the predictive value of ALB. As shown in Figure 1, the Area-Under-the-Curve (AUC) of the Receiver Operating Characteristics (ROC) curve was 0.747, indicating robust predictive ability. Based on the Youden index, the optimal cutoff point for ALB was determined to be 41.45 g/L, which provides the best balance between specificity (64.79%) and sensitivity (85.71%).

## 4. Discussion

In this retrospective study, we identified serum albumin (ALB) as an independent risk factor for intraoperative hypothermia in patients undergoing surgery for HSCR. This finding is significant because it underscores the necessity of monitoring and managing ALB levels in these patients to mitigate the risk of hypothermia during surgery. This discovery offers a fresh foundation for clinical decision-making processes, enabling healthcare providers to take more proactive measures in preventing and managing intraoperative hypothermia.

Previous research endeavours have predominantly centred on conventional surgical factors, such as the duration of the surgery, the type of anaesthesia and the complexity of the surgery [26–28]; however, our comparison of anaesthesia/operation duration between groups revealed no significant differences, likely due to our rigorous thermal care protocol, where active warming measures effectively decoupled procedure length from hypothermia risk. Our study innovatively incorporates the laboratory indicator of ALB into our analysis. This inclusion addresses a notable research gap in this field, as previous studies have largely overlooked the potential role of such biomarkers in intraoperative hypothermia. Consequently, this finding carries significant implications for the perioperative management strategies employed in surgical interventions for HSCR patients. To further elucidate the underlying mechanisms, additional investments in research are deemed necessary.

Albumin is the predominant protein in human plasma, accounting for approximately 60% of the total serum proteins [29]. It is synthesized in the liver and plays a multifaceted role in maintaining the body’s physiological balance. Albumin plays a pivotal role in maintaining oncotic pressure, which is essential for fluid balance in the vascular system [30,31]. Maintaining this balance is crucial for ensuring the adequate distribution of bodily fluids and preventing the accumulation of abnormal fluids. Albumin is a highly versatile protein involved in the transportation of a range of both endogenous and exogenous compounds, including fatty acids essential for energy metabolism, hormones regulating physiological processes and drugs used to treat numerous diseases [28]. Additionally, it modulates endothelial cell function, thereby enhancing microcirculation [28,31–33].

Despite the well-established functions of albumin, the current understanding regarding the influence of albumin on intraoperative hypothermia remains inconclusive [25]. Surgical trauma primarily produces a local acute inflammatory reaction and triggers a series of inflammatory cytokine storms [34,35]. The cytokine response to injury increases the permeability of the capillary membrane to proteins, and thereby albumin is redistributed from the intravascular to the interstitial space, leading to the decrease in serum albumin concentration [36]. Hypoalbuminemia also correlates with overhydration caused by a rapid intravenous infusion during surgery [37,38]. Hypoalbuminemia-induced fluid imbalance can lead to changes in circulatory volume, thereby reducing blood flow efficiency [39]. Blood acts as a heat carrier, helping to distribute the heat generated by metabolic processes throughout the body. When circulatory volume is insufficient, the efficiency of heat distribution is reduced, making it difficult to maintain a stable internal temperature [40]. Additionally, fluid imbalance can affect the thermoregulatory function of blood vessels [41]. Capillaries play a role in regulating heat [42,43]. When the body needs to conserve heat, blood vessels constrict, reducing blood flow to the skin and preventing heat loss [41]. However, in cases of insufficient blood volume, the efficiency of vasoconstriction is reduced, weakening the body’s ability to conserve heat [40]. Moreover, fluids are essential for cellular metabolism and energy production, and energy is the primary source of heat. When cells lack sufficient fluid, metabolic processes slow down, reducing heat production and impairing the body’s ability to maintain the core temperature.

Our research has uncovered a significant correlation between albumin levels and intraoperative hypothermia in HSCR patients. This observation underscores the importance of monitoring serum albumin levels during preoperative assessments for HSCR patients. Future studies should endeavour to establish a definitive causal relationship between albumin levels and intraoperative temperature regulation, potentially inform updated guidelines for perioperative care in at-risk patient populations. This endeavour will facilitate healthcare providers in more accurately anticipating and mitigating the occurrence of intraoperative hypothermia. We also identified 41.45 g/L as the optimal threshold of ALB for predicting intraoperative hypothermia, with a specificity of 64.79% and a sensitivity of 85.71%. Preoperatively, patients with low ALB levels might receive nutritional support to enhance the body’s stress tolerance. During the surgery, the nurses might prepare these patients effective warming equipment and measures, including application of external heat to skin and peripheral tissues, interventions to promote heat retention, and warming intravenous fluids to body temperature. Postoperatively, greater emphasis should be placed on monitoring and warming of patients. The clinical decision-making process, grounded in more precise correlations, might enable the provision of more individualized and efficacious perioperative care for high-risk patient cohorts.

There are still several limitations inherent in this study. Firstly, the modest sample size employed restricts the generalizability of our findings, potentially limiting their applicability to broader populations. Secondly, the study’s single-centre design may hinder the extrapolation of our results to other settings, thereby limiting their universal applicability. Thirdly, the absence of data pertaining to intraoperative hypothermia patients represents a notable gap in our analysis. To address these limitations and validate our conclusions, future research endeavours should strive for larger sample sizes, incorporate multicentre studies, and employ prospective cohort designs. Furthermore, investigating the potential of ALB as a biomarker necessitates a deeper dive into patients with diverse underlying conditions and those undergoing varying surgical procedures. By elucidating the role of albumin in these contexts, researchers can gain valuable insights into its correlation with inflammatory responses and surgical outcomes. Moreover, exploring the intricate interactions between albumin and other inflammatory biomarkers could enhance our comprehension of the inflammatory processes that impact surgical patients [44]. Ultimately, this line of research holds the potential to inform clinical practices, leading to the development of improved strategies for monitoring and managing perioperative care. By optimizing patient outcomes across a spectrum of surgical conditions, such research can contribute significantly to the advancement of surgical care.

## 5. Conclusion

In summary, this study identified ALB as an independent risk factor for intraoperative hypothermia in patients diagnosed with HSCR. This association remains consistent across various models with diverse adjustments. These findings offer new insights into intraoperative hypothermia research and might inform future prevention and management strategies for HSCR patients.

## Data Availability

The datasets used and analysed in the current study are available from the corresponding authors Junjie Wang and Donghao Guo upon reasonable request.
